# Evolution of Climatic Related Leaf Traits in the Family Nothofagaceae

**DOI:** 10.3389/fpls.2018.01073

**Published:** 2018-07-27

**Authors:** Nataly Glade-Vargas, Luis F. Hinojosa, Marcelo Leppe

**Affiliations:** ^1^Laboratorio de Paleoecología, Facultad de Ciencias, Universidad de Chile, Santiago, Chile; ^2^Instituto de Ecología y Biodiversidad, Santiago, Chile; ^3^Instituto Antártico Chileno, Punta Arenas, Chile

**Keywords:** evolution, paleoclimate, phylogenetic signal, Nothofagaceae, leaf physiognomy

## Abstract

The current relationship between leaf traits and environmental variables has been widely used as a proxy for climate estimates. However, it has been observed that the phylogenetic relationships between taxa also influence the evolution of climatic related leaf traits, implying that the direct use of the physiognomy–climate relation should be corrected by their ancestor–descendant relations. Here, we analyze the variation of 20 leaf traits during the evolution of 27 species in the Gondwana family Nothofagaceae. We evaluate whether the evolution of these traits is exclusively associated with past climate variations or whether they are restricted by phylogenetic relationships. Our results indicate that four leaf traits, associated with size and shape, had consistently a phylogenetic independent evolution, suggesting adaptive variation with the environment. While three of the traits, presented consistently phylogenetic signal and fit a Brownian motion or Ornstein-Uhlenbeck model of evolution, suggesting that the evolution of these traits is restrained by phylogenetic relationships and implying that phylogenetic corrections should be made for the family Nothofagaceae to use them as climatic proxy. Finally, this study highlights the importance of evaluating the evolutionary history of climatic related leaf traits before conducting paleoclimate estimates.

## Introduction

The genus *Nothofagus* (Nothofagaceae) has been considered a key taxon for understanding the present distribution pattern and the evolution of the Southern Hemisphere flora (Tanai, 1986; Linder and Crisp, 1995; Manos, 1997). The genus *Nothofagus* has 35 species, with four monophyletic subgenera: *Nothofagus, Fuscospora, Lophozonia*, and *Brassospora* (Hill and Read, 1991). Recently, Heenan and Smissen (2013) proposed to update the rank of the four groups to genera, given the morphological and molecular differences between them. But, as Hill et al. (2015) argued, this classification update would induce confusion between the modern taxonomy and the fossil records, which represents a large proportion of the family taxa.

At present the genus has a wide and disjunct distribution in the temperate regions of the Southern Hemisphere. The subgenus *Nothofagus* is endemic to South America; *Lophozonia* is distributed in South America, New Zealand, Australia, and Tasmania; *Fuscospora* is distributed in New Zealand, Tasmania, and South America. *Brassospora*, which is the genus found at lower latitudes, is distributed in the islands of Papua New Guinea (and associated islands) and New Caledonia (Dettmann et al., 1990; Manos, 1997).

It has been established from palynological records that the genus had a wider distribution in western Gondwana 80 Mya (Dettmann et al., 1990). During the Cenozoic Era, the four subgenera were found in South America, Australia, Tasmania, and New Zealand, places where Nothofagaceae is currently distributed. However, subgenus *Brassospora* reached its current distribution during the Miocene via long distance dispersion or expansion of its distribution range when these landmasses emerged from the ocean (Coleman, 1980; Romero, 1986; Dettmann et al., 1990; Hill and Dettmann, 1996; Hope, 1996; Hinojosa et al., 2016).

Regarding the climatic niche of Nothofagaceae, the subgenus *Brassospora* has the highest values of Mean Annual Temperature (MAT) and Annual Precipitation (AP) (20.8°C and 3,229.5 mm, respectively). Subgenus *Nothofagus* has an average MAT of 7.6°C and AP of 1,526 mm; *Fuscospora* has an average MAT of 7.6°C and AP of 1,640 mm; and *Lophozonia* has an average MAT of 10.9°C and AP of 1,347 mm (Hinojosa et al., 2016).

Leaf morphology has wide variation within the family Nothofagaceae, highlighting the contrast between the temperate forms with respect to those of tropical latitudes (Romero, 1980). The species that are distributed in Papua New Guinea, and New Caledonia have entire leaf margins, or exceptionally have a slightly serrate margin (*N. discoidea* and *N. balansae*); their leaf length ranges from 4 to 12 cm (Van Steenis, 1953). In contrast, leaves of the temperate species have non-entire margins with simple or compound teeth and leaf length of 1 to 3.5 cm, with the exception of *N. solandri* and *N. cliffortioides*, which have entire leaf margin and leaf length of 1 to 1.2 cm (Ogden et al., 1996).

Leaf morphology and climate relation has been the subject of several studies since Bailey and Sinnott (1915, 1916) analyzed for the first time the relation between the proportion of leaves of woody dicotyledonous species with entire leaf margin and MAT along a latitudinal gradient. These authors described a global pattern where the tropical and subtropical flora presented a higher proportion of species with entire leaf margin, while cold temperate flora presented a higher proportion of leaves with non-entire margin. Different methodologies have been developed after this pioneering study, both univariate and multivariate, allowing the study of the leaf physiognomy–climate relation, such as Leaf Margin Analysis and CLAMP (Wolfe, 1971, 1979, 1985, 1993). These physiognomic analyses have been especially productive for paleoclimatic reconstructions (Hinojosa and Villagrán, 2005; Royer et al., 2005; Hinojosa et al., 2006, 2011, 2016; Peppe et al., 2011) and are made under the assumption that the leaf physiognomy–climate relationship is independent of phylogeny. However, Hinojosa et al. (2011) found that the percentage of entire leaf margin of Chilean flora has a high phylogenetic signal and is strongly correlated with its phytogeographic elements. Likewise, Little et al. (2010) found that the historical factor was the major driver of the distribution of the current linages of the southeast United States and that the decrease in temperature favored the diversification of those linages.

The fossil records of Nothofagaceae show that their leaf morphology has exhibited low variation through time, suggesting high levels of conservation during the evolution of these species (Hill, 1991). Hinojosa and Villagrán (2005) indicated that traits associated with climate in fossils of *Nothofagus* presented a wider multivariate range than the current ones, implying that the climatic range of fossils was broader than the current species, and by extension leaf traits were subjected to stabilizing selection through their evolutionary history.

Given the importance of the family Nothofagaceae in the biogeographic history of the current flora, in this study we ask the following question: Did leaf traits that are associated with climate evolve independently from the phylogenetic history of the family? If so, the evolution of leaf traits should fit a phylogeny-independent model of evolution, like a White Noise Model (WN) of evolution, and present low phylogenetic signal or even absence of it. Conversely, if leaf traits are restrained by phylogenetic relationships, we expect that these traits should fit a Brownian Motion Model (BM) or an Ornstein-Uhlenbeck Model (OU) of evolution along with the presence of phylogenetic signal. To address this question, we measured 20 leaf traits from digital leaf photographs of Nothofagaceae species to test the phylogenetic signal (Pagel’s lambda) of these traits, their fit to three alternative models of evolution (BM, WN, and OU) and their Phylogenetic Signal Representation curves.

## Materials and Methods

### Image Processing and Leaf Measurements

**FIGURE 1:**
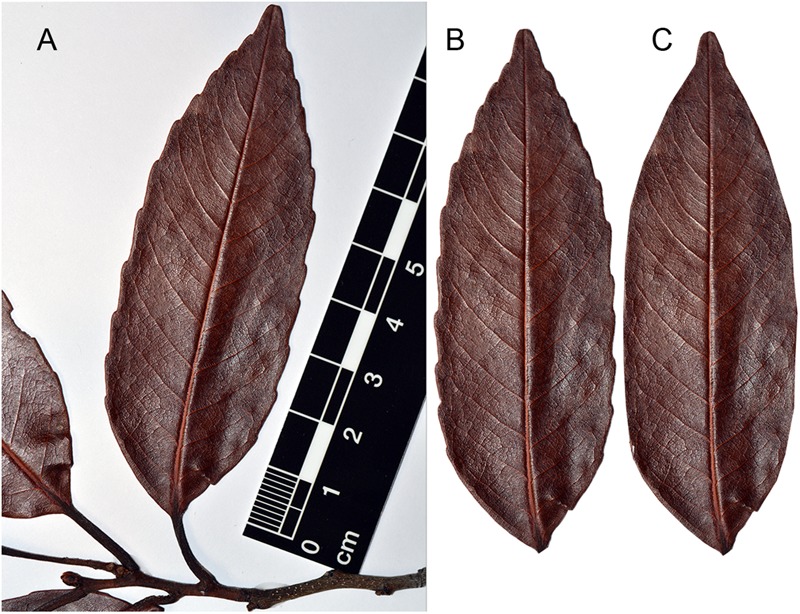
Preparation of the digital leaf samples. **(A)** Original image of *N. discoidea* (*Brassospora*) with scale. **(B)** Leaf sample with petiole removed. **(C)** Leaf with teeth removed.

We used 2,340 leaf samples of 27 species of Nothofagaceae obtained from regional herbaria records collected since 1853 (MEL, National Herbarium of Victoria; HO, Tasmanian Herbarium and CON, Universidad de Concepción) and fieldwork stored in the Paleoecology Laboratory of the Universidad de Chile. Each of the 2,340 samples were photographed and from each one we extracted five to six leaves. The petiole and teeth, if present, were removed (**Figure 1**). If the leaf was incomplete, the lamina was filled to eliminate those blank pixels and avoid error in trait measures. Leaves that were severely damaged were not used. The processing of the images was done with Adobe^®^ Photoshop^®^ 6.0 (Adobe Systems Inc., San Jose, CA, United States). The size of our sampling should be enough for accounting between species variation and detect site-level patterns, as computerized resampling has shown (Royer et al., 2005; Peppe et al., 2011).

We obtained measures of 20 leaf traits established by Royer et al. (2005), associated with the size, shape and teeth of the leaf (**Table 1**). Images and measurements were made using Sigma Scan Pro^®^ (SPSS Science, Chicago, IL, United States) following the protocol of Huff et al. (2003). The output measurements used were area, perimeter, shape factor, Feret diameter, major axis length and minor axis length. Tooth counting was done manually since there is no software that can discriminate teeth from the leaf blade. Tooth area for the toothed species was obtained by the subtraction of the blade area (**Figure 1B**) and the area of leaf in which teeth were previously removed (**Figure 1C**). Untoothed leaves were assigned a tooth area of zero. The rest of the measurements are derived from the traits already mentioned. For the later analyses, we made a logarithmic transformation of the mean values for each trait [Ln(x+1)] to improve normality and homogeneity of data.

**Table 1:** Definitions of leaf traits used in this study (Royer et al., 2005).

Variables	Definition (units)
Blade area, A	Area of leaf blade (cm^2^)
Perimeter, P	Blade perimeter (cm)
Internal Perimeter, Pi	Perimeter after teeth are removed (cm)
Perimeter Ratio, Pratio	Perimeter/internal perimeter (dimensionless)
Compactness, Comp	Perimeter^2^/blade area (dimensionless)
Shape factor, ShapFact	4π × Blade area/perimeter^2^ (dimensionless)
Major axis length, MajLen	Longest measurable line across leaf blade (cm)
Minor axis length, MinLen	Longest measurable line perpendicular to major axis (cm)
Feret diameter, FerDiam	Diameter of circle with the same area as leaf (cm)
Feret diameter ratio, FerDiamRatio	Feret diameter/major axis length (dimensionless)
Tooth area, TA	Area of teeth (cm^2^)
Tooth area:blade area, TABA	(dimensionless)
Tooth area:perimeter, TA:P	(cm)
Tooth area:internal perimeter, TA:Pi	(cm)
Number of primary teeth, 1° teeth	(count)
Number of secondary teeth, 2° teeth	(count)
Number of teeth, #teeth	Number of primary and secondary teeth
Average tooth area, AvgTA	Tooth area/number of primary teeth (cm^2^)
Number of teeth:perimeter, #teeth:P	(cm^-1^)
Number of teeth:internal perimeter, #teeth:Pi	(cm^-1^)

To evaluate how leaf traits are correlated between each other, we obtained a Pearson correlation matrix using the *corrplot* function from the R package corrplot (Wei and Simko, 2016), using R v. 3.3.3 (R Core Team, 2017).

Most of these traits have been previously associated with climatic conditions. Blade area correlates positively with AP, MAT and with others derived moisture and temperature variables related to growing season. While tooth area and number of teeth correlate negatively with MAT, Shape factor and compactness, traits that are associated with the circularity of the blade, correlate positively with it (Huff et al., 2003; Royer et al., 2005, 2009a,b; Royer and Wilf, 2006; Peppe et al., 2011; Schmerler et al., 2012; Wright et al., 2017). Although, it has been observed that the strength of these correlations is greater in woody species than in non-woody species (Wright et al., 2017).

Therefore, these traits should be good proxies to test if the evolution of leaf morphology has been restrained by climate during the evolution of these taxa.

### Phylogenetic Signal and Comparative Analyses

To evaluate the evolution of leaf traits in Nothofagaceae we used the maximum clade credibility BEAST tree topology published by Sauquet et al. (2012), calibrated with macro and micro fossil records (Chronogram scenario 4, Figure 3 in Sauquet et al., 2012). To test the phylogenetic signal of leaf traits we estimated Pagel’s lambda (Pagel, 1994), which ranges from zero to one. Lambda (λ) is a scaling parameter for the correlations between species, so λ equal to zero means there is no correlation between species and that trait evolution is independent of phylogeny. Conversely, λ equal to one means there is a correlation between species equal to the Brownian expectation, and thus trait evolution is strongly influenced by phylogeny (Pagel, 1999). The λ parameter was estimated for each leaf trait using the *phylosig* function from the R-package Phytools (Revell et al., 2012). To evaluate the fit of each trait we used the Akaike Information Criterion for three evolutionary models: (1) a BM model of gradual and continuous drift, (2) an OU model which can be thought of as a stabilizing selection model of evolution with one optimum, and (3) a WN model of random variation, in which the variation of the trait is independent of phylogenetic relationships (Hansen et al., 2008; Hawkins et al., 2014). The fitting to these alternative models was made using the *fitContinuous* function from the R package Geiger (Harmon et al., 2008). All the analyses were made with R v. 3.3.3 (R Core Team, 2017).

### Phylogenetic Signal Representation Curve

We constructed a Phylogenetic Signal Representation curve (PSR curve, Diniz-Filho et al., 2012) to evaluate the model of evolution that best fit leaf trait variation. This curve is constructed by successive extraction of Eigenvectors from the distance matrix that describes the phylogenetic relationships among species. These Eigenvectors are used successively as explanatory variables in a standard ordinary least squares (OLS) regression to model trait variation. The PSR curve is constructed by plotting the coefficients of determination extracted from the regression against the cumulative of eigenvalues. This analysis is comparable to the phylogenetic signal test, since the 45° line of the PSR curve defines a Brownian evolution. The area below the 45° line suggests that traits are evolving slower than expected under a BM model of evolution and have lower phylogenetic signal. It has been described that in this scenario, the evolutionary model that could explain this pattern would be one of stabilizing selection like the OU model of evolution, because this model considers a selection force that is restraining the variation of the trait into ancestral states and thus, lowering the phylogenetic signal (Martins et al., 2002; Ackerly, 2009; Bini et al., 2014). On the contrary, if trait variations reflect an early diversification followed by a stability between species divergence, the amount of phenotypic variation would be greater than expected under a BM scenario, generating PSR curves over the 45° line. This analysis was made with PVR and PSR function from the R-package PVR (Diniz-Filho et al., 2012).

## Results

### Leaf Traits

**FIGURE 2:**
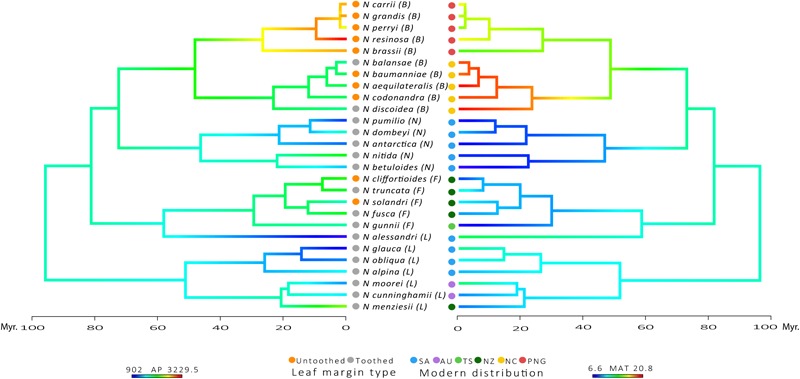
Plot of Leaf margin type character at the tips of the phylogenetic tree along with reconstruction states onto internal edges and nodes of AP and MAT. Modern distribution: SA, South America; AU, Australia; TS, Tasmania; NZ, New Zealand; NC, New Caledonia; PNG, Papua-New Guinea. Subgenera: L, *Lophozonia*; F, *Fuscospora*; N, *Nothofagus*; B, *Brassospora*. Time scale of divergence for Nothofagaceae species.

Species belonging to the tropical subgenus *Brassospora* have the largest leaves in the family, while the temperate subgenus *Nothofagus* has the smallest leaves (A, P, MajLen, MinLen and P_i_; Supplementary Table 1). Subgenus *Nothofagus* also has the most circular leaves, along with the subgenus *Fuscospora*, as indicated by the shape factor index and Feret diameter ratio near to one (ShapeFact and FeretDiamRatio; Supplementary Table 1). Subgenus *Lophozonia* has the largest tooth area and greatest number of primary teeth (TA, TA:BA, TA:P, and TA:P_i_, # teeth and 1° teeth; in Supplementary Table 2). However, subgenus *Nothofagus* has more teeth per blade perimeter and a greater number of secondary teeth (2° teeth, # teeth:P and # teeth:P_i_; Supplementary Table 2). This pattern agrees with the expectations of smaller and toothier leaves in cold temperate climates, as shown in the plot of leaf margin type along with the reconstruction of MAT and AP in the phylogenetic tree of the family (**Figure 2**). Despite the log-transformation of mean values reduce variability of data, outliers can still be observed for some of the leaf traits: TA, TA:BA, TA:P, TA:Pi, and AvgTA (Supplementary Figure 1).

### Phylogenetic Signal and Comparative Analyses

**FIGURE 3:**
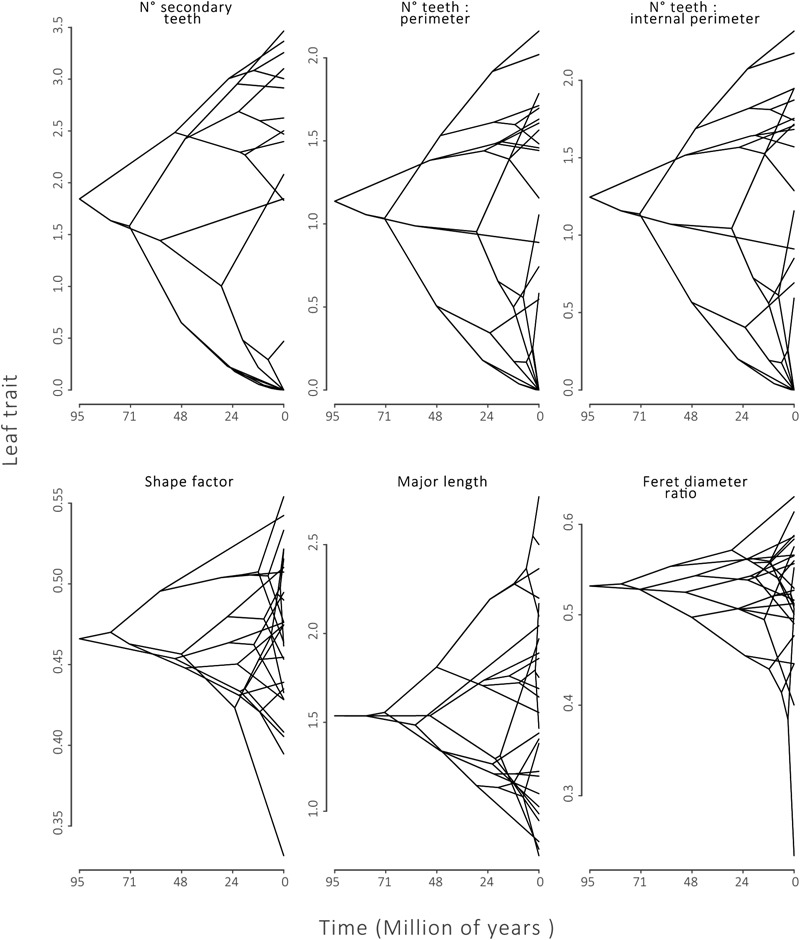
Phylogenetic Traitgrams for leaf traits with strong phylogenetic signal:number of secondary teeth (2° teeth), number of teeth:perimeter and number of teeth:internal perimeter. And for traits that did not present phylogenetic signal: shape factor, minor axis length, Feret diameter. Tips of phylogenies along the *Y*-axis show species trait values. The *X*-axis defines time of divergence from the common ancestor of Nothofagaceae.

Fourteen of the 20 leaf traits presented a phylogenetic signal, indicated by λ significantly different from zero (*p* ≤ 0.01, **Table 2**). Of these traits, 2° teeth, #teeth:P and #teeth:P_i_ had λ equal or near to one, indicating a strong phylogenetic signal. These results can also be observed in a traitgram of these traits, where the pattern of the tree after the trait reconstruction maintained the topology of the phylogenetic tree (**Figure 3**). The rest of the leaf traits presented λ that were not significantly different from zero: Compac, ShapeFact, MinLen, FeretDiamRatio, AvgTA, and TA:P_i_, suggesting that the evolution of these traits is mostly independent of the effect of ancestor–descendant relationships (**Table 2** and **Figure 3**).

**Table 2:** Results of Phylogenetic signal (Pagel’s lambda) and Weighted Akaike, based on exp(-0.5 × ΔAIC), to compare the best fit between a Brownian Motion (BM) model, an Ornstein-Uhlenbeck (OU) model and a White Noise (WN) null model of evolution.

Leaf traits	wAIC			Phylogenetic signal	
	BM	OU	White	λ	*P*
A	0.00	0.40	0.60	0.62	0.01
P	0.03	0.96	0.01	0.74	<0.01
Compac	0.00	0.24	0.76	0.09	0.69
ShapeFact	0.00	0.32	0.68	0.20	0.54
MajLen	0.02	0.97	0.01	0.73	<0.01
MinLen	0.00	0.30	0.70	0.42	0.12
FeretDiam	0.00	0.48	0.52	0.62	0.01
P_i_	0.01	0.86	0.14	0.67	<0.01
Pratio	0.34	0.66	0.00	1.00	<0.01
FerDiamRatio	0.00	0.23	0.77	0.24	0.26
TA	0.19	0.81	0.00	0.80	<0.01
TA:BA	0.47	0.53	0.00	0.80	<0.01
TA:P	0.28	0.72	0.00	0.74	<0.01
TA:P_i_	0.12	0.87	0.01	0.35	0.17
1° teeth	0.00	0.90	0.10	0.54	<0.01
2° teeth	0.78	0.22	0.00	1.00	<0.01
# teeth	0.04	0.96	0.00	0.67	<0.01
AvgTA	0.08	0.83	0.08	0.23	0.19
# teeth:P	0.61	0.39	0.00	0.89	<0.01
# teeth:P_i_	0.63	0.37	0.00	0.89	<0.01

*Please see description of each physiognomic variable in **Table 1**.*

In the fitting of the three alternative evolutionary models, eleven of the twenty leaf traits fit better to an OU model of evolution (wAIC, **Table 2**). Three leaf traits associated with teeth fit better to a BM model of evolution: 2° teeth, # teeth:P and # teeth:P_i_. Six leaf traits associated with size and shape fit better to the null model (WN): A, Compac, ShapeFact, MinLen, FeretDiam, and FeretDiamRatio. These results agree with those obtained for the phylogenetic signal test.

When we perform the phylogenetic signal test and comparative fitting for minimum trait values, we obtain that 16 of the 20 traits evaluated adjust better the WN null model of evolution and 10 of them have not phylogenetic signal (*p*-values > 0.05, wAIC; Supplementary Table 3). For maximum trait values, we obtain that 14 of the 20 traits have not phylogenetic signal and 12 of them adjust better to a WN model of evolution (*p*-values > 0.05, wAIC; Supplementary Table 3).

Still, 2° teeth, #teeth:P and #teeth:Pi are consistently conserved in the family, for both minimum and maximum values, given by lambda values near to one and the best fit to a BM model of evolution, just as observed with the mean values (**Table 2** and Supplementary Table 3, respectively).

### Phylogenetic Signal Representation Curve

**FIGURE 4:**
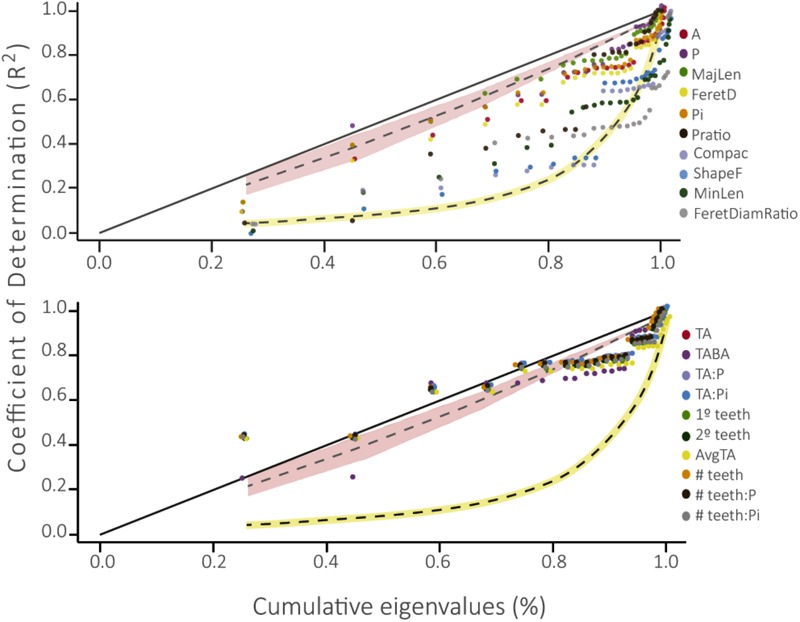
Phylogenetic Signal Representation curve (PSR) for the 10 leaf traits evaluated in this work. The yellow curve represents the White Noise null model of evolution and the pink area below the 45° line represents the Brownian motion neutral model of evolution. Above: PSR curve for traits associated to size and shape of the leaf. Below: PSR curve for traits associated to leaf teeth (please see description of each physiognomic variable in **Table 1**).

Leaf traits associated with size and shape differed from the BM model of evolution and the WN null model of evolution, showing slower evolution than the expectation of a Brownian model (**Figure 4**). However, four of these leaf traits did not differ from the null expectation: Compac, ShapeFact, MinLen, and FeretDiamRatio. Conversely, leaf traits associated with teeth did not differ from the BM model, as these curves tended to adjust to the BM area.

When we perform the PSR curves with the minimum and maximum trait values for each species, we obtained similar patterns. The PSR curves for both maximum and minimum values of traits associated to size and shape of the leaf (Supplementary Figure 2) reflect a model of evolution with phylogenetic signal (PSR curves fall on or under the Brownian expectation) and PSR curves of ShapeFact, Compac, Pratio, and FerDiamRatio reflect an absence of it (PSR curves are not different from the null expectation). Traits associated to number of leaf teeth seems to be conserved in the family, as the PSR curves adjust to the Brownian expectation (Supplementary Figure 2). But, contrarily to what we obtained from mean values, PSR curves for both minimum and maximum values of traits associated to tooth size (TA, TA:BA, TA:P, TA:Pi and AvgTA) are not different from the null model of evolution, indicating a phylogenetically independent evolution for these traits.

## Discussion

### Leaf Traits

We evaluate the pattern of evolution for 20 leaf traits that were previously correlated to climatic variables. We measure seven physiognomic variables: laminar area, perimeter, compactness, shape factor, Feret diameter, and tooth count. From these, we obtained the latter 13 physiognomic variables, as it shows in **Table 1**. Since most of these variables by definition correlates with another one, we expected that the autocorrelated variables behave the same way in our analyses. Within 16 of the 20 leaf traits evaluated we have three clusters of autocorrelation: leaf size and shape related traits, size of leaf teeth related traits and number of leaf teeth related traits (Supplementary Figure 3). Within the last four leaf traits, Shapes factor correlates negatively with compactness and both Feret diameter ratio and perimeter ratio are not autocorrelated. But, since our analyses were made independently for each foliar trait, the effect of the interactions between these traits is discarded.

### Phylogenetic Signal and Evolutionary Models

The phylogenetic signal test showed that most foliar traits evaluated have phylogenetic signal (λ *≠ 0, p* ≤ 0.01; **Table 2**). Notably, the number of secondary teeth, number of teeth: perimeter and number of teeth: internal perimeter had the highest λ values (1.0, 0.88, and 0.89, respectively) and fit better to a BM model of evolution (**Table 2**), suggesting strong conservation of these traits in the family Nothofagaceae (Losos, 2008; Ackerly, 2009). The rest of the traits that presented phylogenetic signal (λ ≠ 0, *p* ≤ 0.01) fit better to an OU model of evolution, except for blade area and Feret diameter, which fit better to the null model. The PSR curve analyses show the same pattern where size and shape traits present phylogenetic signal adjusting to a BM model of evolution, indicating that species inherit their leaf traits from ancestors and slowly diverge as result of neutral drift (**Figure 4**) (Diniz-Filho et al., 2012; Bini et al., 2014). And traits associated to leaf teeth follow an OU model of evolution, suggesting that these traits are constrained to an optimum and evolved slower than the other ones (Martins et al., 2002; Ackerly, 2009).

Finally, the six traits that did not presented phylogenetic signal, Compactness, shape factor, Feret diameter ratio, and minor axis length consistently showed phylogeny-independent evolution, given by the absence of phylogenetic signal and fit to a null model of evolution. Thus, the variation in these traits is independent of phylogenetic relationships between species and could be directly responding to climate variation during the evolutionary history of the Nothofagaceae. In fact, leaf circularity (shape factor and Feret diameter ratio) has been shown to differ within the same species relative to light availability. Salinas (2016) evaluated intra-specific leaf morphology and found that canopy leaves were more circular than those from the understory. Royer et al. (2009b) found that leaves from *Acer rubrum* that originated in colder climate were more dissected (compactness and perimeter ratio) than those from a warmer climate. It has also been established that leaf size is highly variable within the same species, mainly related to incident sunlight, temperature, and precipitation (Ackerly et al., 2002; Knight and Ackerly, 2003; Royer et al., 2005, 2009b; Rozendaal et al., 2006). Indeed, Ackerly et al. (2002) found a significative difference between specific leaf area of sun leaves of *Prunus ilicifolia* that were collected at high and low insolation gradient along east–west ridge tops transect. Royer et al. (2005) found that for intra-specific samples, leaf area increase along with temperature. Therefore, the variation of these traits appears to be strongly related to leaf economics, independently responding to different conditions of light, heat, and moisture (Wright et al., 2004).

Considering that we use all available samples from laboratory collections and from museum herbaria, we did not have control on light exposure or life stage of the individuals sampled. Hence, we expect an important variation in traits measurements within each species, which could affect the results obtained by mean values (Supplementary Tables 1, 2). Nevertheless, for minimum and maximum trait values we observed the same pattern on exception of traits associated to tooth size (TA, TA:BA, TA:P, TA:Pi, and AvgTA). This may be explained by the constant presence of outliers within our measurements (Supplementary Figure 1). Nonetheless, the results reported here do not differ from those obtained using Sauquet et al. (2012) phylogenetic tree from scenario 1 (results not shown), in which calibrations were made using only macrofossils age constraints, supporting the robustness of our results.

### Leaf Morphology Evolution in Nothofagaceae

It appears that the presence of an entire leaf margin in Nothofagaceae represents a singularity and the general rule for the family has been to have a toothed leaf margin (Hill, 1991). Hinojosa et al. (2011) showed that the presence of teeth on leaves of the subgenus *Nothofagus* is strongly restrained by historical factors rather than climatic effects. Additionally, it has been established that Nothofagaceae has low variation in leaf morphology (Romero, 1984; Romero and Dibbern, 1985; Romero, 1986; Dettmann et al., 1990), hence the entire leaf margin is an exception in the family Nothofagaceae. Subgenus *Brassospora*, almost all of whose species have entire leaf margin, coexisted with the rest the Nothofagaceae species with toothed margin in Antarctica and South America, and colonized tropical areas belatedly when the decrease in temperature began (Romero, 1986; Dettmann et al., 1990; Hill and Dettmann, 1996; Hope, 1996; Hinojosa et al., 2016). Indeed, the current distribution of the subgenus is considered as recent, due to the fossil records found on New Caledonia and Papua New Guinea from the Quaternary and Late Miocene, respectively (Dettmann et al., 1990; Hill, 1991; Swenson et al., 2000; Paull and Hill, 2003; Heads, 2006). Therefore, *Brassospora* must have lost or reduced its teeth as its distribution reached lower latitudes. As a matter of fact, the fossil record of *Nothofagus palustris* (subg. *Brassospora*) of the Late Oligocene from New Zealand (Carpenter et al., 2014) had a regularly serrate margin with one or two teeth per secondary vein, indicating that *Brassospora* in the past had a toothed margin like the rest of Nothofagaceae species. There are another two Nothofagaceae species with entire leaf margin, *N. solandri* and *N. cliffortioides* (subg. *Fuscospora*), with leaf size comparable to the other temperate species, but these two *Fuscospora* species are distributed in an environmental space with high precipitation, higher than the precipitation recorded for the rest the temperate species. Thus, this condition could be the driver of the change in margin type for these two species.

## Conclusion

In this work, we ask if climatic related leaf traits on the family Nothofagaceae are restrained by their ancestor–descendant relationships or whether if they are restrained by climatic variables. Our results show that three of the 20 foliar traits evaluated in this study, associated with the number of teeth, presented consistently a high phylogenetic signal and fit a Brownian motion type model of evolution, suggesting that the evolution of the margin type is restrained by phylogenetic relationships in the family Nothofagaceae. Four leaf traits, associated with the size and shape of the leaf, had evolution consistently independent of the phylogeny (compactness, shape factor, Feret diameter ratio, and minor axis length), suggesting adaptive variation with the environment. Thus, for these traits should be phylogenetically corrected for the family Nothofagaceae to be used in paleoclimate estimate. Finally, this study highlights the importance of evaluating the evolutionary history of climatic related leaf traits before conducting paleoclimate estimates.

## Author Contributions

NG-V, LFH, and ML: conception and design of study. NG-V and LFH: acquisition of data, analysis and/or interpretation of data, and drafting and revising the manuscript.

## Conflict of Interest Statement

The authors declare that the research was conducted in the absence of any commercial or financial relationships that could be construed as a potential conflict of interest.

## References

[B1] Ackerly D. (2009). Conservatism and diversification of plant functional traits: evolutionary rates versus phylogenetic signal. *Proc. Natl. Acad. Sci. U.S.A.* 106(Suppl. 2) 19699–19706. 10.1073/pnas.0901635106 19843698 PMC2780941

[B2] Ackerly D. Knight C. Weiss S. Barton K. Starmer K. (2002). Leaf size, specific leaf area and microhabitat distribution of chaparral woody plants: contrasting patterns in species level and community level analyses. *Oecologia* 130 449–457. 10.1007/s004420100805 28547053

[B3] Bailey I. W. Sinnott E. W. (1915). A botanical index of Cretaceous and Tertiary climates. *Science* 41 831–834. 10.1126/science.41.1066.831 17835989

[B4] Bailey I. W. Sinnott E. W. (1916). The climatic distribution of certain types of angiosperm leaves. *Am. J. Bot.* 3 24–39. 10.2307/2435109

[B5] Bini L. M. Villalobos F. Diniz-Filho J. A. F. (2014). Explorando patrones en rasgos macroecológicos utilizando regresión secuencial de autovectores filogenéticos. *Ecosistemas* 23 21–26. 10.7818/ECOS.2014.23-1.04

[B6] Carpenter R. J. Bannister J. M. Lee D. E. Jordan G. J. (2014). Nothofagus subgenus *Brassospora* (Nothofagaceae) leaf fossils from New Zealand: a link to Australia and New Guinea? *Bot. J. Linn. Soc.* 174 503–515. 10.1111/boj.12143

[B7] Coleman P. J. (1980). Plate tectonics background to biogeographic development in the southwest Pacific over the last 100 million years. *Palaeogeogr. Palaeoclimatol. Palaeoecol.* 31 105–121. 10.1016/0031-0182(80)90016-4

[B8] Dettmann M. E. Pocknall D. T. Romero E. J. Zamaloa M. C. (1990). *Nothofagidites* Erdtman ex Potonié, 1960; a catalogue of species with notes on the paleogeographic distribution of *Nothofagus* Bl. (southern beech). *N. Z. Geol. Surv. Paleontol. Bull.* 60 1–79.

[B9] Diniz-Filho F. Alexandre J. Rangel T. F. Santos T. Mauricio Bini L. (2012). Exploring patterns of interspecific variation in quantitative traits using sequential phylogenetic eigenvector regressions. *Evolution* 66 1079–1090. 10.1111/j.1558-5646.2011.01499.x 22486690

[B10] Hansen T. F. Pienaar J. Orzack S. H. (2008). A comparative method for studying adaptation to a randomly evolving environment. *Evolution* 62 1965–1977. 10.1111/j.1558-5646.2008.00412.x 18452574

[B11] Harmon L. J. Weir J. T. Brock C. D. Glor R. E. Challenger W. (2008). GEIGER: investigating evolutionary radiations. *Bioinformatics* 24 129–131. 10.1093/bioinformatics/btm538 18006550

[B12] Hawkins B. A. Rueda M. Rangel T. F. Field R. Diniz- Filho J. A. F. (2014). Community phylogenetics at the biogeographical scale: cold tolerance, niche conservatism and the structure of North American forests. *J. Biogeogr.* 41 23–38. 10.1111/jbi.12171 24563577 PMC3920643

[B13] Heads M. (2006). Panbiogeography of *Nothofagus* (Nothofagaceae): analysis of the main species massings. *J. Biogeogr.* 33 1066–1075. 10.1111/j.1365-2699.2006.01479.x

[B14] Heenan P. B. Smissen R. D. (2013). Revised circumscription of *Nothofagus* and recognition of the segregate genera *Fuscospora, Lophozonia*, and *Trisyngyne* (Nothofagaceae). *Phytotaxa* 146 1–31. 10.11646/phytotaxa.146.1.1

[B15] Hill R. S. (1991). Tertiary *Nothofagus* (Fagaceae) macrofossils from Tasmania and Antarctica and their bearing on the evolution of the genus. *Bot. J. Linn. Soc.* 105 73–112. 10.1111/j.1095-8339.1991.tb00200.x

[B16] Hill R. S. Dettmann M. E. (1996). “Origin and diversification of the genus *Nothofagus*,” in *The Ecology and Biogeography of Nothofagus Forest* eds Veblen T. T. Hill R. S. Read J. (New Haven, CT: University of Yale Press).

[B17] Hill R. S. Jordan G. J. Macphail M. K. (2015). Why we should retain *Nothofagus* sensu lato. *Aust. Syst. Bot.* 28 190–193. 10.1071/SB15026

[B18] Hill R. S. Read J. S. (1991). A revised infrageneric classification of *Nothofagus* (Fagaceae). *Bot. J. Linn. Soc.* 105 37–72. 10.1111/j.1095-8339.1991.tb00199.x

[B19] Hinojosa L. Villagrán C. (2005). Did South American Mixed Paleofloras evolve under thermal equability or in the absence of an effective Andean barrier during the Cenozoic? *Palaeogeogr. Palaeoclimatol. Palaeoecol.* 217 1–23. 10.1016/j.palaeo.2004.11.013

[B20] Hinojosa L. F. Armesto J. J. Villagrán C. (2006). Are Chilean coastal forests pre-Pleistocene relicts? Evidence from foliar physiognomy, palaeoclimate, and phytogeography. *J. Biogeogr.* 33 331–341. 10.1111/j.1365-2699.2005.01350.x

[B21] Hinojosa L. F. Gaxiola A. Pérez M. F. Carvajal F. Campano M. F. Quattrocchio M. (2016). Non-congruent fossil and phylogenetic evidence on the evolution of climatic niche in the Gondwana genus *Nothofagus*. *J. Biogeogr.* 43 555–567. 10.1111/jbi.12650

[B22] Hinojosa L. F. Perez F. Gaxiola A. Sandoval I. (2011). Historical and phylogenetic constraints on the incidence of entire leaf margins: insights from a new South American model. *Glob. Ecol. Biogeogr.* 20 380–390. 10.1111/j.1466-8238.2010.00595.x

[B23] Hope G. S. (1996). *History of Nothofagus in New Guinea and New Caledonia. The Ecology and Biogeography of Nothofagus Forests.* New Haven, CT: Yale University Press 257–270.

[B24] Huff P. M. Wilf P. Azumah E. J. (2003). Digital future for paleoclimate estimation from fossil leaves? Preliminary results. *Palaios* 18 266–274. 10.1669/0883-1351(2003)018<0266:DFFPEF>2.0.CO;2

[B25] Knight C. A. Ackerly D. D. (2003). Evolution and plasticity of photosynthetic thermal tolerance, specific leaf area and leaf size: congeneric species from desert and coastal environments. *New Phytol.* 160 337–347. 10.1046/j.1469-8137.2003.00880.x 33832168

[B26] Linder H. P. Crisp M. D. (1995). *Nothofagus* and Pacific biogeography. *Cladistics* 11 5–32. 10.1111/j.1096-0031.1995.tb00002.x 34920593

[B27] Little S. A. Kembel S. W. Wilf P. (2010). Paleotemperature proxies from leaf fossils reinterpreted in light of evolutionary history. *PLoS One* 5:e15161. 10.1371/journal.pone.0015161 21203554 PMC3008682

[B28] Losos J. B. (2008). Phylogenetic niche conservatism, phylogenetic signal and the relationship between phylogenetic relatedness and ecological similarity among species. *Ecol. Lett.* 11 995–1003. 10.1111/j.1461-0248.2008.01229.x 18673385

[B29] Manos P. (1997). Systematics of *Nothofagus* (Nothofagaceae) based on rDNA spacer sequences (ITS): taxonomic congruence with morphology and plastid sequences. *Am. J. Bot.* 84 1137–1137. 10.2307/2446156 21708668

[B30] Martins E. P. Diniz-Filho J. A. F. Housworth E. A. (2002). Adaptive constraints and the phylogenetic comparative method: a computer simulation test. *Evolution* 56 1–13. 10.1111/j.0014-3820.2002.tb00844.x 11913655

[B31] Ogden J. Steward G. Allen R. (1996). “Ecology of New Zeland *Nothofagus* forest,” in *The Ecology and Biogeography of Nothofagus Forest* eds Veblen T. T. Hill R. S. Read J. (New Haven, CT: University of Yale Press).

[B32] Pagel M. (1994). Detecting correlated evolution on phylogenies: a general method for the comparative analysis of discrete characters. *Proc. R. Soc. Lond. B Biol. Sci.* 255 37–45. 10.1098/rspb.1994.0006

[B33] Pagel M. (1999). Inferring the historical patterns of biological evolution. *Nature* 401 877–884. 10.1038/44766 10553904

[B34] Paull R. Hill R. S. (2003). *Nothofagus kiandrensis* (Nothofagaceae subgenus *Brassospora*), a new macrofossil leaf species from Miocene sediments at Kiandra, New South Wales. *Aust. Syst. Bot.* 16 549–559. 10.1071/SB02033

[B35] Peppe D. J. Royer D. L. Cariglino B. Oliver S. Y. Newman S. Leight E. (2011). Sensitivity of leaf size and shape to climate: global patterns and paleoclimatic applications. *New Phytol.* 190 724–739. 10.1111/j.1469-8137.2010.03615.x 21294735

[B36] R Core Team (2017). *R: A Language and Environment for Statistical Computing.* Vienna: R Foundation for Statistical Computing.

[B37] Revell L. J. Mahler D. L. Peres-Neto P. R. Redelings B. D. (2012). A new phylogenetic method for identifying exceptional phenotypic diversification. *Evolution* 66 135–146. 10.1111/j.1558-5646.2011.01435.x 22220870

[B38] Romero E. J. (1980). Arquitectura foliar de las especies sudamericanas de *Nothofagus* Bl. *Bol. Soc. Argent. Bot.* 19 289–308.

[B39] Romero E. J. (1984). “Historia y evolución de *Nothofagus* (Fagaceae) y consideraciones sobre el origen de otras familias relacionadas,” in *Actas del III Congreso Argentino de Paleontología y Bioestratigrafia* Trelew 209–216.

[B40] Romero E. J. (1986). Paleogene phytogeography and climatology of South America. *Ann. Mo. Bot. Gard.* 73 449–461. 10.2307/2399123

[B41] Romero E. J. Dibbern M. C. (1985). A review of the species described as *Fagus* and *Nothofagus* by Dusen. *Palaeontographica* 197 123–137.

[B42] Royer D. L. Kooyman R. M. Little S. A. Wilf P. (2009a). Ecology of leaf teeth: a multi-site analysis from an Australian subtropical rainforest. *Am. J. Bot.* 96 738–750. 10.3732/ajb.0800282 21628229

[B43] Royer D. L. Meyerson L. A. Robertson K. M. Adams J. M. (2009b). Phenotypic plasticity of leaf shape along a temperature gradient in *Acer rubrum*. *PLoS One* 4:e7653. 10.1371/journal.pone.0007653 19893620 PMC2764093

[B44] Royer D. L. Wilf P. (2006). Why do toothed leaves correlate with cold climates? Gas exchange at leaf margins provides new insights into a classic paleotemperature proxy. *Int. J. Plant Sci.* 167 11–18. 10.1086/497995

[B45] Royer D. L. Wilf P. Janesko D. A. Kowalski E. A. Dilcher D. L. (2005). Correlations of climate and plant ecology to leaf size and shape: potential proxies for the fossil record. *Am. J. Bot.* 92 1141–1151. 10.3732/ajb.92.7.1141 21646136

[B46] Rozendaal D. M. A. Hurtado V. H. Poorter L. (2006). Plasticity in leaf traits of 38 tropical tree species in response to light; relationships with light demand and adult stature. *Funct. Ecol.* 20 207–216. 10.1111/j.1365-2435.2006.01105.x

[B47] Salinas F. (2016). *Variación Fisionómica Foliar y Clima en un Gradiente Latitudinal en Chile.* Tésis de Magíster, Universidad de Chile Santiago.

[B48] Sauquet H. Ho S. Y. W. Gandolfo M. A. Jordan G. J. Wilf P. Cantrill D. J. (2012). Testing the impact of calibration on molecular divergence times using a fossil-rich group: the case of *Nothofagus* (Fagales). *Syst. Biol.* 61 289–313. 10.1093/sysbio/syr116 22201158

[B49] Schmerler S. B. Clement W. L. Beaulieu J. M. Chatelet D. S. Sack L. Donoghue M. J. (2012). Evolution of leaf form correlates with tropical–temperate transitions in *Viburnum* (Adoxaceae). *Proc. R. Soc. Lond. B Biol. Sci.* 279 3905–3913. 10.1098/rspb.2012.1110 22810426 PMC3427575

[B50] Swenson U. Hill R. S. McLoughlin S. (2000). Ancestral area analysis of *Nothofagus* (Nothofagaceae) and its congruence with the fossil record. *Aust. Syst. Bot.* 13 469–478. 10.1071/SB99010

[B51] Tanai T. (1986). Phytogeographic and phylogenetic history of the genus *Nothofagus* BL. (Fagaceae) in the Southern Hemisphere. *J. Fac. Sci. Hokkaido Univ.* 21 505–582.

[B52] Van Steenis C. G. G. J. (1953). Results of the archbold expeditions. Papuan *Nothofagus*. *J. Arnold Arbor.* 34 301–374. 10.5962/bhl.part.27154

[B53] Wei T. Simko V. (2016). *Package ‘corrplot’. R package version* 0.77. Available at: https://CRAN.R-project.org/package=corrplot

[B54] Wolfe J. A. (1971). Tertiary climatic fluctuations and methods of analysis of tertiary floras. *Palaeogeogr. Palaeoclimatol. Palaeoecol.* 9 27–57. 10.1016/0031-0182(71)90016-2

[B55] Wolfe J. A. (1979). *Temperature Parameters of Humid to Mesic Forests of Eastern Asia and Relation to Forests of Other Regions of The Northern Hemisphere and Australasia* Vol. 1106 Washington, DC: US. Geological Survey Professional Paper 1–37.

[B56] Wolfe J. A. (1985). “Distribution of major vegetational types during the Tertiary,” in *The Carbon Cycle and Atmospheric C0_2_. Natural Variations Archean to Present: American Geophysical Union Monograph 32* eds Sundquist E. T. Broecker W. S. (Washington, DC: American Geophysical Union) 357–376.

[B57] Wolfe J. A. (1993). A method of obtaining climatic parameters from leaf assemblages. Bulletin 2040–2041. Washington, DC: U.S. Government Printing Office.

[B58] Wright I. J. Dong N. Maire V. Prentice I. C. Westoby M. Díaz S. (2017). Global climatic drivers of leaf size. *Science* 357 917–921. 10.1126/science.aal4760 28860384

[B59] Wright I. J. Reich P. B. Westoby M. Ackerly D. D. Baruch Z. Bongers F. (2004). The worldwide leaf economics spectrum. *Nature* 428 821–827. 10.1038/nature02403 15103368

