# Supplementary material for: Evolution of Climatic Related Leaf Traits in the Family Nothofagaceae
Source: Front Plant Sci. 2018 Jul 27;9:1073. doi: 10.3389/fpls.2018.01073 (PMC6073098; doi:10.3389/fpls.2018.01073)
Supplement: Supplementary file 1 [file Data_Sheet_1.pdf]

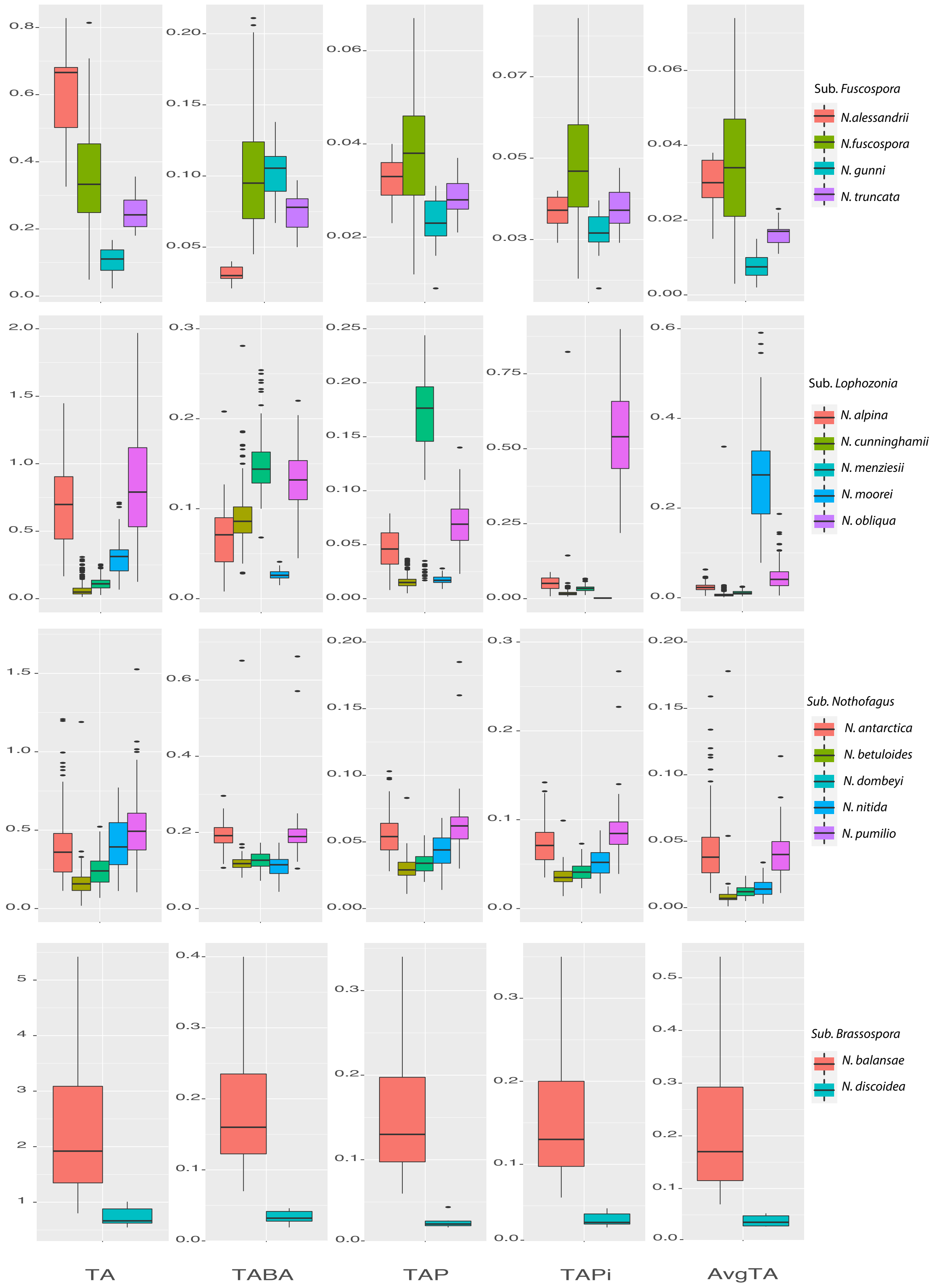

Supplementary Figure 1. Box-plot of traits associated to leaf tooth size for the Nothofagaceae species evauated in this work. Species with entire leaf margin were ommitted (please see description of each physiognomic variable in Table 1).

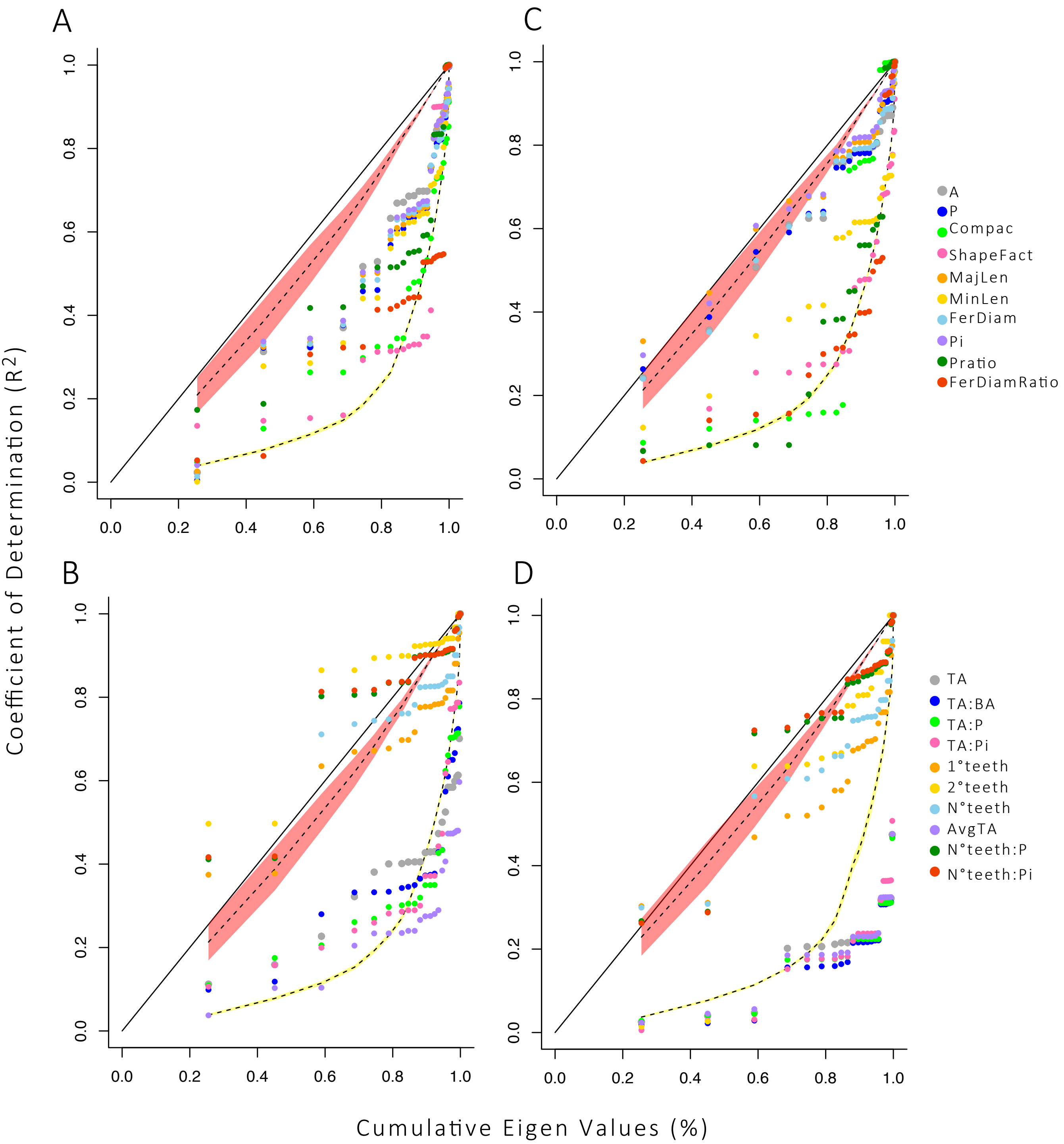

Supplementary Figure 2. Phylogenetic Signal Representation Curves (PSR) for both maximum and minimum values of the 20 leaf traits evaluated in this work. A: PSR curve for maximum values of size and shape related leaf traits. B: PSR curve for minimum values of size and shape related leaf traits. C: PSR curve for maximum values of leaf teeth related traits. D: PSR curve for minimum values of leaf teeth related traits.The yellow curve represents the White Noise null model of evolution and the pink area below the 45º line represents the Brownian Motion neutral model of evolution (please see description of each physiognomic variable in Table 1).

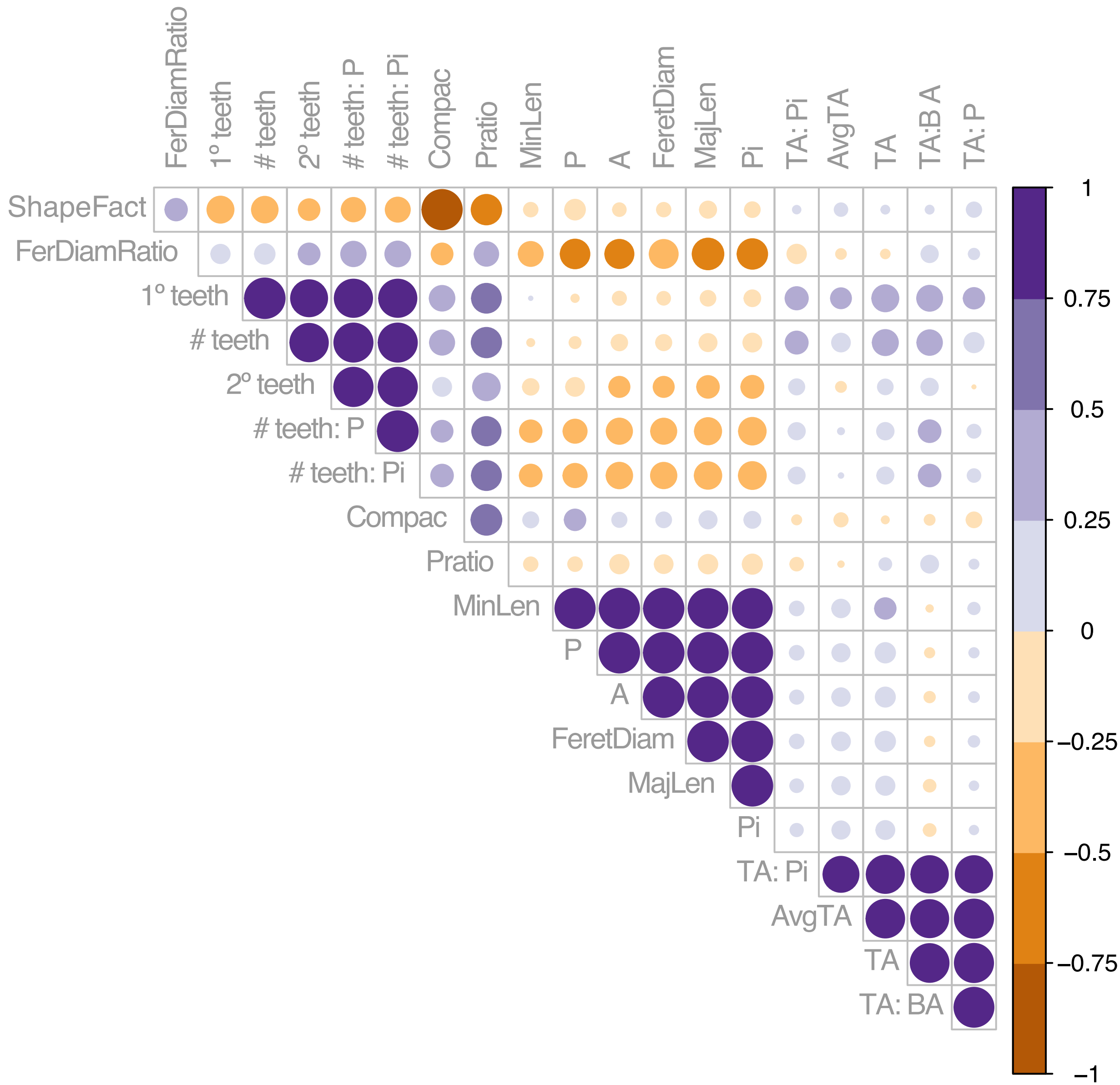

Supplementary Figure 3. Correlations between the 20 leaf traits used in this work (please see description of each physiognomic variable in Table 1).

Supplementary Table 1. Physiognomic variables associated with size and shape of the leaf for the 27 Nothofagaceae species used in this study Mean values and standard deviation values between parenthesis (please see description of each physiognomic variable in Table 1).

Species	A	P	Compac	ShapeF	MajLen	MinLen	FeretD	Pi	Pratio	FeretDiamRatio
Nothofagus menziesii	0.75 (0.26)	4.11 (0.89)	22.94 (2.87)	0.57 (0.08)	1.15 (0.21)	0.94 (0.19)	0.97 (0.17)	3.20 (0.61)	1.28 (0.09)	0.84 (0.05)
Nothofagus cunninghamii	0.73 (0.49)	3.84 (1.45)	22.29 (8.38)	0.61 (0.16)	1.20 (0.46)	0.86 (0.27)	0.92 (0.30)	3.25 (1.10)	1.18 (0.22)	0.79 (0.09)
Nothofagus moorei	12.34 (6.14)	17.22 (5.11)	25.41 (3.63)	0.50 (0.07)	6.17 (1.93)	2.79 (0.67)	3.84 (1.00)	14.93 (4.34)	1.15 (0.05)	0.63 (0.06)
Nothofagus alpina	11.86 (7.12)	15.07 (4.17)	20.86 (2.39)	0.61 (0.07)	5.62 (1.63)	2.68 (0.82)	3.74 (1.06)	13.69 (4.04)	1.11 (0.08)	0.67 (0.04)
Nothofagus obliqua	7.42 (4.02)	12.86 (4.13)	24.17 (5.41)	0.54 (0.11)	4.42 (1.32)	2.19 (0.64)	2.94 (0.83)	10.62 (3.08)	0.93 (0.45)	0.49 (0.26)
Nothofagus glauca	14.91 (9.97)	16.95 (6.27)	20.86 (5.27)	0.64 (0.12)	5.42 (1.90)	3.44 (1.03)	4.17 (1.28)	14.22 (4.73)	1.19 (0.14)	0.78 (0.04)
Nothofagus alessandrii	20.91 (8.68)	19.14 (5.67)	17.87 (3.57)	0.72 (0.10)	6.72 (1.64)	4.24 (0.83)	5.07 (1.01)	18.17 (4.27)	1.04 (0.05)	0.76 (0.06)
Nothofagus gunni	1.08 (0.54)	4.42 (1.08)	18.69 (4.10)	0.66 (0.07)	1.29 (0.34)	1.07 (0.28)	1.14 (0.30)	3.68 (0.99)	0.83 (0.04)	0.88 (0.03)
Nothofagus fusca	3.68 (1.72)	9.12 (2.16)	24.39 (3.58)	0.53 (0.07)	2.72 (0.73)	1.89 (0.47)	2.10 (0.53)	7.23 (1.87)	1.27 (0.10)	0.78 (0.04)
Nothofagus solandri	0.99 (0.52)	3.99 (1.12)	17.05 (1.14)	0.74 (0.05)	1.58 (0.49)	0.81 (0.19)	1.09 (0.28)	3.99 (1.12)	1.00 (0.00)	0.70 (0.05)
Nothofagus truncata	3.34 (0.64)	8.73 (1.20)	22.96 (2.81)	0.56 (0.07)	2.79 (0.49)	1.81 (0.16)	2.05 (0.20)	7.19 (0.94)	1.21 (0.05)	0.75 (0.07)
Nothofagus cliffortioides	0.44 (0.11)	2.78 (0.36)	17.88 (0.89)	0.70 (0.03)	1.12 (0.14)	0.53 (0.08)	0.74 (0.09)	2.78 (0.36)	1.00 (0.00)	0.66 (0.03)
Nothofagus betuloides	1.51 (1.10)	5.36 (1.46)	20.84 (1.71)	0.61 (0.05)	1.79 (0.49)	1.11 (0.30)	1.32 (0.35)	4.49 (1.22)	1.19 (0.05)	0.74 (0.05)
Nothofagus nitida	3.87 (1.61)	9.40 (2.33)	23.77 (4.03)	0.53 (0.08)	3.22 (0.75)	1.83 (0.44)	2.17 (0.47)	7.86 (1.85)	1.19 (0.10)	0.68 (0.08)
Nothofagus antarctica	2.11 (1.14)	6.77 (1.93)	23.25 (3.26)	0.55 (0.07)	2.00 (0.55)	1.41 (0.39)	1.58 (0.41)	5.14 (1.39)	1.32 (0.09)	0.80 (0.06)
Nothofagus dombeyi	1.97 (0.83)	7.09 (1.67)	26.59 (4.07)	0.48 (0.07)	2.40 (0.51)	1.18 (0.28)	1.55 (0.33)	5.73 (1.22)	1.23 (0.08)	0.65 (0.05)
Nothofagus pumilio	2.77 (1.24)	8.19 (2.76)	25.34 (7.94)	0.54 (0.15)	2.31 (0.74)	1.68 (0.55)	1.87 (0.60)	5.94 (1.92)	1.44 (0.43)	0.85 (0.24)
Nothofagus discoidea	23.34 (6.99)	28.06 (5.25)	35.59 (12.78)	0.39 (0.14)	9.64 (1.46)	3.77 (0.82)	5.41 (0.75)	22.25 (3.28)	1.27 (0.22)	0.56 (0.01)
Nothofagus codonandra	21.88 (13.39)	21.17 (7.79)	22.46 (4.16)	0.57 (0.08)	8.19 (2.67)	3.28 (1.15)	5.02 (1.66)	21.17 (7.79)	1.00 (0.00)	0.61 (0.04)
Nothofagus aequilateralis	11.74 (3.87)	16.43 (3.51)	23.57 (3.22)	0.54 (0.07)	6.81 (1.51)	2.21 (0.60)	3.81 (0.70)	16.43 (3.51)	1.00 (0.00)	0.57 (0.04)
Nothofagus baumanniae	42.83 (7.53)	29.12 (2.80)	19.91 (0.91)	0.63 (0.03)	11.19 (1.11)	5.14 (0.56)	7.36 (0.65)	29.12 (2.80)	1.00 (0.00)	0.66 (0.02)
Nothofagus balansae	12.19 (1.43)	14.95 (0.99)	18.36 (0.35)	0.68 (0.01)	5.77 (0.30)	2.77 (0.24)	3.94 (0.23)	14.57 (0.76)	1.03 (0.01)	0.68 (0.01)
Nothofagus brassii	4.53 (0.72)	9.22 (0.74)	18.90 (0.58)	0.67 (0.02)	3.74 (0.33)	1.58 (0.18)	2.39 (0.19)	9.22 (0.74)	1.00 (0.00)	0.64 (0.02)
Nothofagus resinosa	6.73 (2.21)	12.16 (2.23)	22.87 (5.40)	0.57 (0.12)	4.17 (0.56)	2.10 (0.38)	2.89 (0.47)	12.16 (2.23)	1.00 (0.00)	0.69 (0.07)
Nothofagus perryi	22.48 (16.56)	19.36 (7.99)	18.72 (1.32)	0.67 (0.05)	7.74 (3.39)	3.51 (1.18)	5.01 (1.94)	19.36 (7.99)	1.00 (0.00)	0.66 (0.04)
Nothofagus grandis	8.53 (3.93)	13.09 (2.41)	24.66 (12.62)	0.59 (0.17)	4.77 (1.27)	2.26 (0.63)	3.19 (0.87)	13.09 (2.41)	1.00 (0.00)	0.67 (0.07)
Nothofagus carrii	4.82 (1.93)	9.37 (2.13)	18.79 (3.12)	0.68 (0.10)	3.33 (0.79)	1.91 (0.35)	2.44 (0.47)	9.37 (2.13)	1.00 (0.00)	0.74 (0.03)

Supplementary Table 2. Physiognomic variables of traits associated with leaf teeth for the 27 Nothofagaceae species used in this study. Mean values and standard deviation values between parenthesis (please see description of each physiognomic variable in Table 1).

Species	AD	AD/A	AD/P	AD/Pi	Dientes1	Dientes2	ND	ADp	ND/P	ND/Pi
Nothofagus menziesii	0.11 (0.05)	0.15 (0.03)	0.16 (0.06)	0.03 (0.01)	10.21 (1.88)	5.66 (1.81)	15.87 (2.71)	0.01 (0.00)	3.97 (0.79)	5.08 (1.00)
Nothofagus cunninghamii	0.07 (0.12)	0.09 (0.04)	0.02 (0.03)	0.02 (0.04)	8.97 (1.56)	5.24 (1.93)	14.57 (3.39)	0.01 (0.01)	4.11 (1.15)	4.78 (1.32)
Nothofagus moorei	0.31 (0.14)	0.03 (0.01)	0.02 (0.00)	0.00 (0.00)	43.52 (7.91)	11.20 (9.58)	54.70 (14.41)	0.28 (0.13)	3.30 (0.75)	3.81 (0.94)
Nothofagus alpina	0.71 (0.32)	0.07 (0.03)	0.05 (0.02)	0.05 (0.02)	29.76 (7.14)	27.95 (20.89)	57.71 (24.73)	0.02 (0.01)	3.99 (1.72)	4.52 (2.23)
Nothofagus obliqua	0.98 (0.62)	0.14 (0.05)	0.07 (0.04)	0.56 (0.16)	20.51 (3.36)	24.94 (11.81)	45.44 (12.74)	0.05 (0.03)	3.78 (1.41)	4.56 (1.70)
Nothofagus glauca	1.40 (0.92)	0.10 (0.04)	0.08 (0.03)	0.09 (0.04)	17.00 (4.84)	19.19 (15.44)	36.19 (18.16)	0.08 (0.04)	2.18 (0.98)	2.62 (1.34)
Nothofagus alessandrii	0.61 (0.15)	0.03 (0.01)	0.03 (0.01)	0.03 (0.01)	20.78 (2.64)	5.33 (3.24)	26.11 (3.89)	0.03 (0.01)	1.43 (0.35)	1.49 (0.33)
Nothofagus gunni	0.10 (0.04)	0.10 (0.02)	0.02 (0.01)	0.03 (0.01)	13.77 (2.07)	7.00 (3.35)	20.77 (4.67)	0.01 (0.00)	4.96 (1.65)	6.01 (2.09)
Nothofagus fusca	0.35 (0.15)	0.10 (0.04)	0.04 (0.01)	0.05 (0.02)	11.22 (2.74)	2.11 (1.39)	13.32 (2.92)	0.03 (0.02)	1.58 (0.67)	2.01 (0.83)
Nothofagus solandri	0.00 (0.00)	0.00 (0.00)	0.00 (0.00)	0.00 (0.00)	0.00 (0.00)	0.00 (0.00)	0.00 (0.00)	0.00 (0.00)	0.00 (0.00)	0.00 (0.00)
Nothofagus truncata	0.25 (0.06)	0.08 (0.01)	0.03 (0.00)	0.03 (0.01)	15.74 (1.63)	3.26 (2.31)	19.00 (3.40)	0.02 (0.00)	2.19 (0.32)	2.66 (0.42)
Nothofagus cliffortioides	0.00 (0.00)	0.00 (0.00)	0.00 (0.00)	0.00 (0.00)	0.00 (0.00)	0.00 (0.00)	0.00 (0.00)	0.00 (0.00)	0.00 (0.00)	0.00 (0.00)
Nothofagus betuloides	0.20 (0.38)	0.12 (0.05)	0.03 (0.05)	0.04 (0.06)	21.29 (2.97)	17.46 (5.95)	38.69 (6.48)	0.01 (0.02)	7.68 (2.27)	9.15 (2.75)
Nothofagus nitida	0.42 (0.17)	0.11 (0.03)	0.04 (0.01)	0.05 (0.01)	28.91 (5.91)	30.94 (11.42)	59.79 (13.30)	0.01 (0.01)	6.54 (1.67)	7.80 (2.03)
Nothofagus antarctica	0.40 (0.22)	0.19 (0.03)	0.06 (0.01)	0.07 (0.02)	9.07 (1.91)	21.22 (9.53)	30.29 (9.87)	0.05 (0.03)	4.54 (1.07)	6.00 (1.59)
Nothofagus dombeyi	0.24 (0.09)	0.13 (0.02)	0.03 (0.01)	0.04 (0.01)	20.42 (4.72)	10.82 (4.75)	31.24 (7.94)	0.01 (0.00)	4.45 (0.67)	5.50 (0.95)
Nothofagus pumilio	0.51 (0.22)	0.20 (0.07)	0.06 (0.02)	0.09 (0.03)	13.08 (4.41)	12.81 (4.74)	25.89 (8.66)	0.04 (0.02)	3.40 (1.23)	4.70 (1.76)
Nothofagus discoidea	0.74 (0.19)	0.03 (0.01)	0.03 (0.01)	0.03 (0.01)	19.67 (3.50)	0.00 (0.00)	19.67 (3.50)	0.04 (0.01)	0.72 (0.18)	0.91 (0.23)
Nothofagus codonandra	0.00 (0.00)	0.00 (0.00)	0.00 (0.00)	0.00 (0.00)	0.00 (0.00)	0.00 (0.00)	0.00 (0.00)	0.00 (0.00)	0.00 (0.00)	0.00 (0.00)
Nothofagus aequilateralis	0.00 (0.00)	0.00 (0.00)	0.00 (0.00)	0.00 (0.00)	0.00 (0.00)	0.00 (0.00)	0.00 (0.00)	0.00 (0.00)	0.00 (0.00)	0.00 (0.00)
Nothofagus baumanniae	0.00 (0.00)	0.00 (0.00)	0.00 (0.00)	0.00 (0.00)	0.00 (0.00)	0.00 (0.00)	0.00 (0.00)	0.00 (0.00)	0.00 (0.00)	0.00 (0.00)
Nothofagus balansae	12.19 (15.12)	1.00 (1.27)	0.81 (1.02)	0.83 (1.05)	11.00 (0.82)	0.00 (0.00)	11.00 (0.82)	1.12 (1.35)	0.74 (0.09)	0.76 (0.09)
Nothofagus brassii	0.00 (0.00)	0.00 (0.00)	0.00 (0.00)	0.00 (0.00)	0.00 (0.00)	0.00 (0.00)	0.00 (0.00)	0.00 (0.00)	0.00 (0.00)	0.00 (0.00)
Nothofagus resinosa	0.00 (0.00)	0.00 (0.00)	0.00 (0.00)	0.00 (0.00)	0.00 (0.00)	0.00 (0.00)	0.00 (0.00)	0.00 (0.00)	0.00 (0.00)	0.00 (0.00)
Nothofagus perryi	0.00 (0.00)	0.00 (0.00)	0.00 (0.00)	0.00 (0.00)	0.00 (0.00)	0.00 (0.00)	0.00 (0.00)	0.00 (0.00)	0.00 (0.00)	0.00 (0.00)
Nothofagus grandis	0.00 (0.00)	0.00 (0.00)	0.00 (0.00)	0.00 (0.00)	0.00 (0.00)	0.00 (0.00)	0.00 (0.00)	0.00 (0.00)	0.00 (0.00)	0.00 (0.00)
Nothofagus carrii	0.00 (0.00)	0.00 (0.00)	0.00 (0.00)	0.00 (0.00)	0.00 (0.00)	0.00 (0.00)	0.00 (0.00)	0.00 (0.00)	0.00 (0.00)	0.00 (0.00)

Supplementary Table 3. Results of Phylogenetic signal (Pagel`s Lambda) and Weighted Akaike, based on exp(-0.5 x ΔAIC) to compare the best fit of maximum and minimum physiognomic trait values between a Brownian Motion (BM) model, an Ornstein-Uhlenbeck (OU) model and a White Noise (WN) null model of evolution (please see description of each physiognomic variable in Table 1).

Leaf traits	wAIC						Phylogenetic signal			
	Maximum values			Minimum values			Maximum values		Minimum values	
	**BM**	**OU**	**white**	**BM**	**OU**	**white**	**lambda**	***p-value***	**lambda**	***p-value***
**A**	0.00	0.22	0.78	0.01	0.74	0.25	0.57	0.039	0.68	< 0.01
**P**	0.00	0.22	0.78	0.11	0.86	0.03	0.46	0.079	0.73	< 0.01
**Compac**	0.00	0.22	0.78	0.45	0.46	0.10	0.08	0.731	0.00	1.000
**ShapeFact**	0.03	0.57	0.41	0.00	0.22	0.78	0.03	0.865	0.06	0.749
**MajLen**	0.00	0.22	0.78	0.09	0.88	0.03	0.51	0.054	0.75	< 0.01
**MinLen**	0.00	0.22	0.78	0.00	0.38	0.62	0.40	0.138	0.37	0.123
**FeretDiam**	0.00	0.22	0.78	0.02	0.82	0.16	0.49	0.061	0.70	< 0.01
**Pi**	0.00	0.29	0.71	0.23	0.77	0.01	0.53	0.043	0.80	< 0.01
**Pratio**	0.11	0.82	0.07	0.05	0.65	0.30	0.43	0.071	0.00	1.000
**FerDiamRatio**	0.00	0.56	0.43	0.00	0.33	0.66	0.17	0.528	0.00	1.000
**TA**	0.00	0.25	0.75	0.00	0.22	0.78	0.13	0.509	0.00	1.000
**TA: A**	0.00	0.25	0.75	0.00	0.22	0.78	0.11	0.583	0.00	1.000
**TA: P**	0.00	0.24	0.76	0.00	0.22	0.78	0.06	0.748	0.00	1.000
**TA: Pi**	0.00	0.24	0.76	0.00	0.22	0.78	0.04	0.821	0.00	1.000
**1° teeth**	0.02	0.90	0.09	0.00	0.59	0.41	0.62	0.001	0.46	0.020
**2° teeth**	0.77	0.23	0.00	0.67	0.33	0.00	1.00	< 0.01	1.00	< 0.01
**#teeth**	0.07	0.91	0.02	0.00	0.75	0.24	0.69	< 0.01	0.57	< 0.01
**AvgTA**	0.00	0.23	0.77	0.00	0.22	0.78	0.00	1.000	0.00	1.000
**#teeth: P**	0.64	0.36	0.00	0.25	0.74	0.00	0.89	< 0.01	0.78	< 0.01
**#teeth: Pi**	0.65	0.35	0.00	0.30	0.70	0.00	0.89	< 0.01	0.79	< 0.01
